# Successful treatment of rheumatoid arthritis‐associated interstitial lung disease with filgotinib: A case report on janus kinase 1 inhibition

**DOI:** 10.1002/rcr2.70023

**Published:** 2024-09-08

**Authors:** Atsuhiko Sunaga, Takuya Inoue

**Affiliations:** ^1^ Department of Rheumatology Matsushita Memorial Hospital Moriguchi‐shi Japan

**Keywords:** filgotinib, interstitial lung disease, JAK inhibitor, RA‐associated interstitial lung disease, rheumatoid arthritis

## Abstract

Filgotinib, a janus kinase 1 (JAK1) inhibitor, is used in the treatment of rheumatoid arthritis (RA). RA‐associated interstitial lung disease (RA‐ILD) is a severe RA complication with no established effective treatment. We report the case of a patient with RA‐ILD successfully treated with filgotinib. A 46‐year‐old woman with RA and RA‐ILD, presenting with a non‐specific interstitial pneumonia pattern, was refractory to abatacept and prednisolone but responded to filgotinib. Both arthritis and RA‐ILD improved significantly, and the patient remained in remission for over 12 months. Basic research indicates that JAK1 plays a role in the cytokine signal transduction in ILD; however, there are no clinical reports on the efficacy of filgotinib in RA‐ILD. This case suggests filgotinib as a potential treatment for patients with RA‐ILD, particularly in the early stages of this disease.

## Graphical abstract

A 46‐year‐old woman presented with RA and RA‐ILD. Following filgotinib treatment, sustained remission of arthritis and substantial reduction of interstitial pneumonia were achieved.

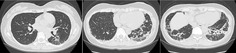

## INTRODUCTION

Interstitial lung disease (ILD) is a severe complication of rheumatoid arthritis (RA), commonly referred to as RA‐ILD, and is associated with poor prognosis. The rates of clinical progression of RA‐ILD vary widely, and effective treatments for this condition remain elusive. Several reports suggest that biologic agents that act as disease‐modifying anti‐rheumatic drugs such as abatacept (ABT), tocilizumab, and rituximab may stabilize RA‐ILD; however, the efficacy of these treatments has not yet been established.^1^ Janus kinase (JAK) inhibitors (JAKis) target intracellular signal transduction in multiple cytokines and could represent a promising strategy for treating RA‐ILD. Filgotinib is an oral small‐molecule inhibitor with JAK1 selectivity.^2^ However, evidence supporting the effectiveness of JAKis, especially filgotinib, in RA‐ILD is limited. This report presents a case of RA associated with ILD with substantial improvement in both RA and RA‐ILD symptoms after treatment with filgotinib.

## CASE REPORT

**FIGURE 1:**
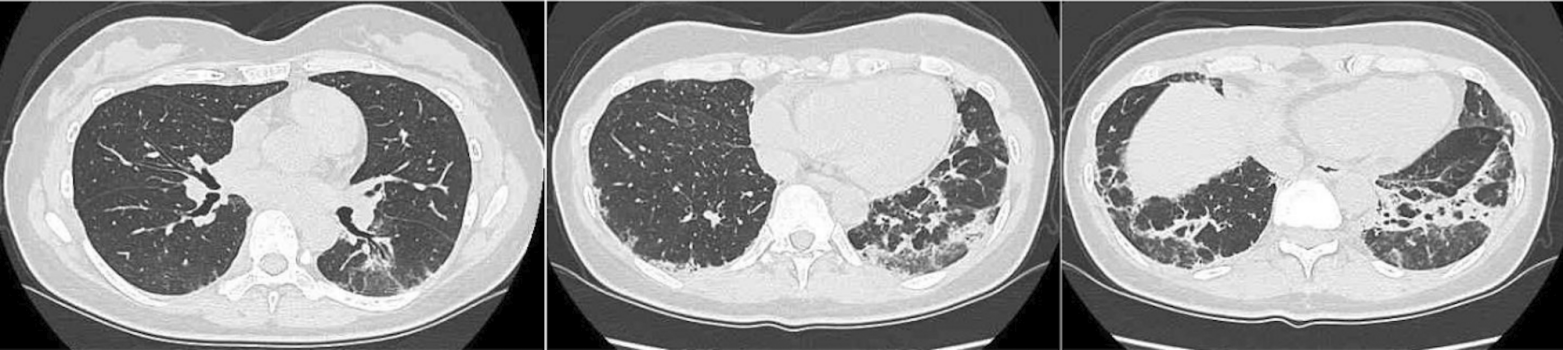
Chest computed tomography during patient's first visit.

**FIGURE 2:**
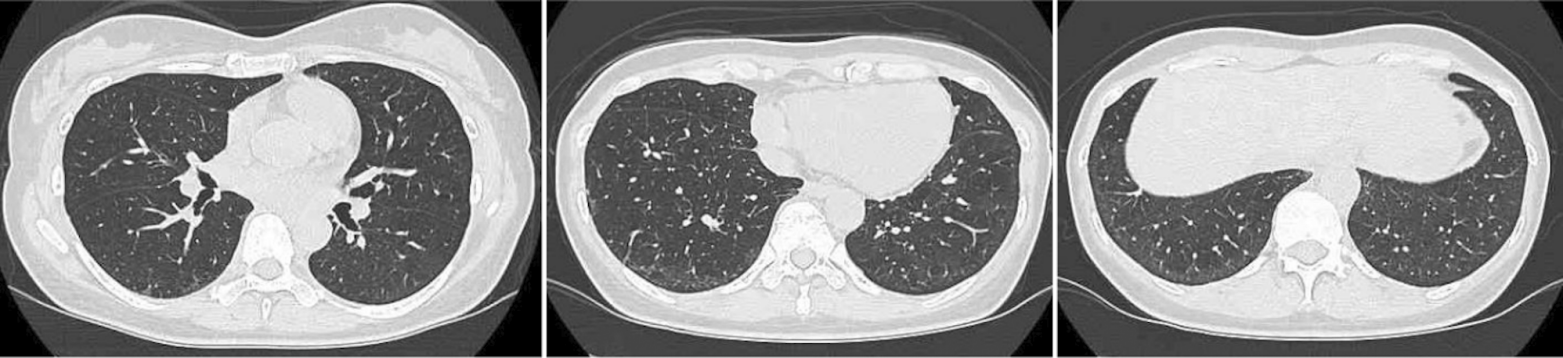
Chest computed tomography 4 months after filgotinib initiation.

A 46‐year‐old Japanese woman presented with a one‐month history of polyarthralgia and a dry cough. She denied experiencing dyspnea. She had no significant medical history and was a non‐smoker. Clinical examination revealed tenderness and swelling in the hand, knee, and proximal interphalangeal joints without evidence of skin rash, muscle stiffness, or weakness. No decrease in SpO2 was observed. Laboratory tests revealed a slight elevation in leukocyte count (9600/μL) predominantly neutrophils (77.4%) and lymphocytes (11.6%), C‐reactive protein (1.04 mg/dL), erythrocyte sedimentation rate (42 mm/h), and Krebs von den Lungen‐6 (565 U/mL). Lactate dehydrogenase and creatine kinase levels were normal. Tests for rheumatoid factor (248 IU/mL) and anti‐cyclic citrullinated peptide antibody (222.8 U/mL) were positive, while those for anti‐nuclear, anti‐aminoacyl‐tRNA synthetase, and anti‐SS‐A antibodies were negative. A chest computed tomography (CT) scan showed extensive consolidations and ground‐glass opacities (GGO) along bronchovascular bundles in the lower lobes, indicative of interstitial pneumonia with features of both organizing pneumonia and non‐specific interstitial pneumonia patterns (Figure 1). After consultation with rheumatologists, respiratory physicians, and radiologists, the patient was diagnosed with both pulmonary and extra‐pulmonary manifestations of RA. Given that her arthritis symptoms were severe while her respiratory condition was mild despite the CT findings, and considering the infection risk associated with RA‐ILD, we decided to start treatment with DMARDs and low‐dose glucocorticoids as needed. She was initially treated with abatacept (125 mg weekly subcutaneous injection) with no improvement in arthritis symptoms. Prednisolone (5 mg/day) was added 4 weeks after initiating ABT, but only partial improvement was noted. Due to the primary failure of ABT, it was replaced with filgotinib (200 mg/day) 8 weeks after initiating ABT. Following the switch, both arthritis symptoms and dry cough improved significantly. Prednisolone tapering began 3 weeks after initiating filgotinib and was discontinued after 5 months. Six months post‐filgotinib initiation, follow‐up chest CT showed a substantial reduction in infiltration, with minimal GGO (Figure 2). RA has remained in remission after discontinuing prednisolone, with no recurrence of RA‐ILD over 12 months.

## DISCUSSION

Our patient presented with acute ILD associated with RA, showing a good response to filgotinib, a selective JAK1 inhibitor. The 2023 American College of Rheumatology /American College of Chest Physicians Guideline recommends mycophenolate, azathioprine, and rituximab for RA‐ILD.^3^ However, these drugs are not covered by insurance for RA patients in Japan, necessitating the selection of alternative treatments.

Numerous cytokines implicated in ILD are components of the JAK/STAT signalling pathway. JAK1 is involved in the signal transduction of various cytokines such as IL‐2, IL‐4, IL‐6, IL‐11, IL‐13, and IFN‐γ, which are either pro‐inflammatory or pro‐fibrotic and associated with ILD.^2^, ^4^ In a bleomycin‐induced fibrosis mouse model, JAK1 is overexpressed in lung tissues and is predominantly localized within inflammatory and epithelial cells.^4^ Although the pathogenesis of RA‐ILD remains unclear and might differ from other types of ILD, these findings underscore the potential therapeutic role of JAK1 inhibitors in managing RA‐ILD. JAK1 also plays a crucial role in the pathology of arthritis, while JAKis are used in treatment of RA: tofacitinib (TOF, JAK1/3 inhibitor); peficitinib (pan JAK inhibitor); baricitinib (BAR, JAK1/2 inhibitor); upadacitinib (JAK1/2 inhibitor); and filgotinib‐target JAK1.^2^

Clinical efficacy of different JAKis for articular and pulmonary manifestations of RA remains unclear. Filgotinib has the highest selectivity for JAK1. While some side effects, such as anaemia, neutropenia, venous thromboembolic events and viral infections, can be attributed to the lack of selectivity in other JAKis for RA, the selectivity of filgotinib may reduce these side effects to some extent.^2^ Although there is no head‐to‐head trial comparing between JAKis, side‐by‐side comparisons suggest that filgotinib might have lower incidence rates of cytopenia, venous thromboembolic events and herpes zoster infection.^5^ Given the high incidence of complication in the treatment of RA‐ILD,^1^ the safety profile of filgotinib might be a factor in distinguishing it from other JAKis.

The effectiveness of the JAK1‐selective inhibitor filgotinib in RA‐ILD remains unknown; however, clinical studies have demonstrated the efficacy of JAKis, particularly TOF and BAR, which are both capable of inhibiting JAK1, in treating RA‐ILD. Moreover, Tardella M et al. retrospectively analysed the extent of fibrotic findings on high‐resolution CT prior to and after treatment with JAKi (31 cases: 13 with TOF and 18 with BAR) and ABT (44 cases).^6^ In the JAKi group, fibrosis improved in 19.4%, remained stable in 64.5%, and progressed in 16.1% of cases. The duration of RA was related to the progression of RA‐ILD in patients treated with JAKis. Similarly, Tsujii A et al. compared the efficacy and safety of JAKi (10 cases with TOF and 16 with BAR) and ABT (45 cases) retrospectively.^7^ They found that the chest CT score for GGO significantly improved in the JAKi group, whereas the scores for GGO and fibrosis did not improve in the ABT group. Although CT findings do not always accurately reflect the underlying pathology, GGO and consolidation could indicate inflammation or infiltration rather than fibrosis in RA‐ILD, suggesting that anti‐inflammatory therapy might be beneficial.^1^ Regarding our case and based on these findings, ILD improvement could be explained by the fact that this disease was in its early inflammatory phase. Our case indicates that filgotinib could be an effective treatment option for RA‐ILD, especially in cases diagnosed in early stage and characterized by GGO and consolidations on chest CT.

In conclusion, when treating RA complicated by RA‐ILD, JAKis should be considered as a potential treatment option. Furthermore, JAK1 inhibition may be beneficial in early RA‐ILD cases associated with GGO on chest CT.

## AUTHOR CONTRIBUTIONS

*Case conceptualization and study design*: Atsuhiko Sunaga. *Data collection and case management*: Atsuhiko Sunaga. *Data analysis and interpretation*: Atsuhiko Sunaga, Takuya Inoue. *Writing – original draft preparation*: Atsuhiko Sunaga. *Writing – review and editing*: Atsuhiko Sunaga, Takuya Inoue. *Supervision*: Takuya Inoue.

## CONFLICT OF INTEREST STATEMENT

Atsuhiko Sunaga has honorariums for lectures from Abbvie. Takuya Inoue has no conflicts of interest.

## ETHICS STATEMENT

The authors declare that appropriate written informed consent was obtained for the publication of this manuscript and accompanying images.

## Data Availability

Data sharing not applicable to this article as no datasets were generated or analysed during the current study.

## References

[1] Yamakawa H , Ogura T , Kameda H , Kishaba T , Iwasawa T , Takemura T , et al. Decision‐making strategy for the treatment of rheumatoid arthritis‐associated interstitial lung disease (RA‐ILD). J Clin Med Res [Internet]. 2021;10(17):3806. 10.3390/jcm10173806 PMC8432201 34501253

[2] Bonelli M , Kerschbaumer A , Kastrati K , Ghoreschi K , Gadina M , Heinz LX , et al. Selectivity, efficacy and safety of JAKinibs: new evidence for a still evolving story. Ann Rheum Dis. 2024;83(2):139–160. 37923366 10.1136/ard-2023-223850 PMC10850682

[3] Johnson SR , Bernstein EJ , Bolster MB , Chung JH , Danoff SK , George MD , et al. 2023 American College of Rheumatology (ACR)/American College of CHEST Physicians (CHEST) guideline for the treatment of interstitial lung disease in people with systemic autoimmune rheumatic diseases. Arthritis Rheumatol [Internet]. 2024;76:1182–1200. 10.1002/art.42861 38978310 PMC12646471

[4] Huo R , Guo Q , Hu J , Li N , Gao R , Mi L , et al. Therapeutic potential of Janus kinase inhibitors for the Management of Interstitial Lung Disease. Drug des Devel Ther. 2022;16:991–998. 10.2147/DDDT.S353494 PMC8985822 35400994

[5] Tanaka Y , Kavanaugh A , Wicklund J , McInnes IB . Filgotinib, a novel JAK1‐preferential inhibitor for the treatment of rheumatoid arthritis: an overview from clinical trials. Mod Rheumatol. 2022;32(1):1–11. 33740386 10.1080/14397595.2021.1902617

[6] Tardella M , Di Carlo M , Carotti M , Ceccarelli L , Giovagnoni A , Salaffi F . A retrospective study of the efficacy of JAK inhibitors or abatacept on rheumatoid arthritis‐interstitial lung disease. Inflammopharmacology. 2022;30(3):705–712. 35462572 10.1007/s10787-022-00936-w PMC9135879

[7] Tsujii A , Isoda K , Yoshimura M , Nakabayashi A , Kim DS , Tamada T , et al. Janus kinase inhibitors vs. abatacept about safety and efficacy for patients with rheumatoid arthritis‐associated interstitial lung disease: a retrospective nested case‐control study. BMC. Rheumatology. 2024;8(1):4. 38273359 10.1186/s41927-024-00374-x PMC10811846

